# Quantitative Proteomic Analysis Reveals That Anti-Cancer Effects of Selenium-Binding Protein 1 *In Vivo* Are Associated with Metabolic Pathways

**DOI:** 10.1371/journal.pone.0126285

**Published:** 2015-05-14

**Authors:** Qi Ying, Emmanuel Ansong, Alan M. Diamond, Zhaoxin Lu, Wancai Yang, Xiaomei Bie

**Affiliations:** 1 College of Food Science and Technology, Nanjing Agricultural University, Nanjing, 210095, China; 2 Department of Pathology, Xinxiang Medical University, Xinxiang, 453003, China; 3 Department of Pathology, University of Illinois at Chicago, Chicago, Illinois 60612, United States of America; China Medical University, TAIWAN

## Abstract

Previous studies have shown the tumor-suppressive role of selenium-binding protein 1 (SBP1), but the underlying mechanisms are unclear. In this study, we found that induction of SBP1 showed significant inhibition of colorectal cancer cell growth and metastasis in mice. We further employed isobaric tags for relative and absolute quantitation (iTRAQ) to identify proteins that were involved in SBP1-mediated anti-cancer effects in tumor tissues. We identified 132 differentially expressed proteins, among them, 53 proteins were upregulated and 79 proteins were downregulated. Importantly, many of the differentially altered proteins were associated with lipid/glucose metabolism, which were also linked to Glycolysis, MAPK, Wnt, NF-kB, NOTCH and epithelial-mesenchymal transition (EMT) signaling pathways. These results have revealed a novel mechanism that SBP1-mediated cancer inhibition is through altering lipid/glucose metabolic signaling pathways.

## Introduction

The human selenium-binding protein 1 (SBP1, SELENBP1) belongs to the class of selenium-containing proteins in which selenium is tightly associated with the peptide instead of incorporated co-translationally as an amino acid selenocysteine (Sec) in another class of selenium-containing protein—glutathione peroxidase 1 (GPx1). The human SBP1 gene encodes a 472 amino acid protein which has a molecular weight equal to 56 KDa. It is expressed in a variety of cells and tissues (liver, heart, lung, and kidney) and located in the nucleus and/or cytoplasm depending on cell type, degree of differentiation, and environmental signaling [1–4]. Decreased SBP1 has been found in most of human cancers including liver cancer, prostate cancer, breast cancer, lung cancer, and colorectal cancer [5]. This reduction of SBP1 is associated with poor clinical outcomes. However, biological functions of SBP1 in human cancers remain unclear.

It has been reported that SBP1 is a target of hypoxia-inducible factor-1 α (HIF1α) to respond to reactive oxygen species (ROS) [3]. SBP1 could also negatively regulate glutathione peroxidase 1 (GPx1) activity without changing its protein expression through a physical protein interaction [6]. von Hippel-Lindau protein (pVHL)- interacting deubiquitinating enzyme 1 (VDU1) was recently found as another protein partner of SBP1, which was involved in ubiquitination- and deubiquitination-mediated protein degradation pathways [7]. All this work suggests that SBP1 may interact with other proteins through different signaling pathways.

To get a more comprehensive understanding of SBP1 functions on cancer, we employed a proteomic method, isobaric tags for relative and absolute quantitation (iTRAQ). iTRAQ is currently one of the most robust techniques which quantifies proteins on the basis of peptide labeling. Unlike gel-based proteomic method, iTRAQ exhibits much better sensitivity and allows the identification and accurate quantification of proteins from multiple samples within broad dynamic ranges of protein abundance [8].

To eliminate possible side effects of transfection, we established a stable SBP1 inducible cell line using a Tet-on inducible gene expression system that makes SBP1 expression highly controllable and reaches a very high induction level in cells. Tumor inhibition role of SBP1 was observed *in vivo*. The differentially expressed proteins in SBP1-induced mouse xenograft tumors were assayed by iTRAQ and the changes of some identified proteins were validated by immunoblotting. Our results revealed a novel anti-cancer mechanism of SBP1 *in vivo*, which was linked to lipid/glucose metabolism and associated with MAPK/Wnt, NF-kB, NOTCH and EMT signaling pathways.

## Materials and Methods

### Ethics Statement

This study was approved by the Xinxiang Medical University Animal Care and Use Committee. All surgery was performed under pentobarbital anesthesia and all efforts were made to minimize suffering.

### Cell culture

The HCT116 human colon carcinoma cell line was obtained from ATCC (American Type Culture Collection, USA) and maintained in McCoy’s 5a medium (Mediatech, Manassas, VA). GP2-293 cell line was purchased from Clontech (Mountain View, CA) and maintained in DMEM medium (Life Technologies, Carlsbad, CA). All media was supplemented with 10% FBS, 100 U/mL penicillin, and 100 μg/mL streptomycin. All cells were cultured at 37°C with 5% CO_2_ in a humidified incubator (Thermo Fisher Scientific).

### Plasmid construction

To generate retroviral expression plasmid pRetroX-Tight-Pur-SBP1, human SBP1 cDNA was amplified and subcloned into the pRetroX-Tight-Pur vector (Clontech, Mountain View, CA) using NotI and EcoRI restriction sites. The following primers were used: forward 5’- ATAGCGGCCGCTACAGCATGGCTACGAAAT-3’ and reverse 5’-ACGAATTCGCTCAAATCCA GATGTCAGAGC-3’. Two plasmids pRetroX-Tet-On Advanced (Tet on activator vector) and pVSV-G (Packaging vector) were purchased from Clontech (Mountain View, CA).

### Retrovirus packaging

The packaging cells GP2-293 were grown in 25cm^2^ flasks and transfected using Lipofectamine 2000 (Life Technologies) with 6 μg pVSV-G and either 2 μg pRetroX-Tight-Pur-SBP1 or 2 μg pRetroX-Tight-On Advanced. Forty-eight hours after transfection, virus containing supernatant was collected and frozen at -20°C. Cell line HCT116-TetSBP1 was generated by infecting HCT116 cells with virus containing both pRetroX-Tight-Pur-SBP1 and pRetroX-Tight-On Advanced. Single colony was picked after selection with 400 μg/mL G418 (Sigma) and 1 μg/mL Puromycin (Sigma). Cell line HCT116-Act was generated by infecting HCT116 cells with virus containing pRetroX-Tight-On Advanced. Single colony was picked after selection with 400 μg/mL of G418 (Sigma).

### Immunoblotting analysis

HCT116-TetSBP1 and HCT116-Act cells were treated with 1μg/mL doxycycline (Sigma) for 72 hours before analysis. Harvested cells were lysed in 1x Cell Lysis Buffer (Cell Signaling, Danvers, MA) containing 1mM PMSF (Cell Signaling). Cleared cell lysate from each cell line was boiled with NuPAGE LDS Sample Buffer (Life Technologies) and 10x Reducing Agent (Life Technologies) for 5 minutes before being loaded on a 4–12% gradient Bis-Tris denaturing polyacrylamide gel (Life Technologies). After sample separation by electrophoresis, proteins were transferred to an Immobilon-FL membrane (Millipore) via electroblotting. Primary antibodies and dilutions including SBP1 at 1:1000 (mouse, MBL International, Woburn, MA), β-actin at 1:10000 (mouse, Sigma), DKK1 at 1:1000 (rabbit, Proteintech, Chicago, IL), ANXA4 at 1:1000 (rabbit, Proteintech, Chicago, IL), PPIA at 1:1000 (rabbit, Proteintech, Chicago, IL), FABP4 at 1:1000 (rabbit, Proteintech, Chicago, IL), UGDH at 1:1000 (rabbit, Proteintech, Chicago, IL), ALDH2 at 1:1000 (rabbit, Proteintech, Chicago, IL), p21 at 1:1000 (rabbit, Proteintech, Chicago, IL), phospho-Akt (Ser473) at 1:1000 (rabbit, Cell Signaling Technology, Danvers, MA), Akt at 1:1000 (rabbit, Cell Signaling Technology, Danvers, MA), phospho-FOXO1 at 1:1000 (rabbit, Cell Signaling Technology, Danvers, MA), phospho-JNK at 1:1000 (rabbit, Cell Signaling Technology, Danvers, MA), JNK at 1:1000 (rabbit, Cell Signaling Technology, Danvers, MA), P-c-Jun at 1:1000 (rabbit, Cell Signaling Technology, Danvers, MA), phospho-Cyclin D1 at 1:1000 (rabbit, Cell Signaling Technology, Danvers, MA), Cyclin D1 at 1:1000 (rabbit, Cell Signaling Technology, Danvers, MA), phospho-p53 (Ser15) at 1:1000 (rabbit, Cell Signaling Technology, Danvers, MA), TWIST at 1:1000 (rabbit, Cell Signaling Technology, Danvers, MA), β–Catenin at 1:500 (goat, Santa Cruz, Santa Cruz, CA), and GPX1 at 1:1000 (mouse, MBL International, Woburn, MA) were incubated with membrane at 4°C overnight. The appropriate secondary antibody (Beyotime Institute of Biotechnology, China) was applied (1:1000, rabbit or mouse) at room temperature for 1 h. Signal was detected using BeyoECL Plus (Beyotime Institute of Biotechnology, China).

### *In vivo* nude mice tumorigenesis and metastasis assay

For xenograft experiment, HCT116-Act and HCT116-TetSBP1 cells were treated with 1μg/mL doxycycline for 72 hours and then 2×10^6^ cells in 100μl PBS were injected subcutaneously into 6–8 weeks-old BALB/c CD-1 Nu female mice on the different sides (HCT116-Act was on the left side and HCT116-TetSBP1 was on the right side). Doxycycline (200 μg/mL) dissolved in 5% sucrose supplied as drinking water was exchanged every 3 days. Four weeks after injection, tumors were isolated and the weight (g) and volume (mm^3^) of tumors were determined. Tumors were stored at -80°C until used for immunoblot and iTRAQ analysis.

To produce experimental lung metastasis, HCT116-Act and HCT116-TetSBP1 cells were treated with 1μg/mL doxycycline for 72 hours and then 2×10^6^ cells in 100 μl PBS were injected into the lateral tail veins of 6–8 weeks-old BALB/c CD-1 Nu female mice. Doxycycline (200 μg/mL) dissolved in 5% sucrose supplied as drinking water was exchanged every 3 days. Six weeks later, mice were sacrificed under anesthesia. The lungs were collected and fixed in 10% formalin. For tissue morphology evaluation, hematoxylin and eosin (HE) staining was performed on sections from embedded samples.

### Protein extraction, digestion and iTRAQ labeling

Total proteins were extracted from tumor tissue of HCT116-Act injected (Act) and HCT116-TetSBP1 injected mice (TetSBP1) using the following protocol. Three biological replicates were carried out for each sample and mixed together for protein extraction. Tumor tissue was boiled in SDT buffer (4%SDS,100mM Tris-HCl,1mM DTT,pH7.6) for 5 min, homogenized using FastPrep-24 (MP Biomedicals, 6M/S, 30s, two cycles) and then sonicated (100w, 30 sec on, 30 sec off, 10 cycles). The homogenate was centrifuged at 12,000g for 10 min at 4°C. Protein concentrations were assayed according to Bradford method [9]. An equal amount of proteins was prepared for each sample. Protein digestion was performed according to the FASP procedure described by Wisniewski, Zougman et al. [10] and the resulting peptide mixture was labeled using the 4-plex iTRAQ reagent according to the manufacturer’s instructions (Applied Biosystems). Briefly, 200 μg of proteins for each sample were incorporated into 30 μl STD buffer (4% SDS, 100 mM DTT, 150 mM Tris-HCl pH 8.0). The detergent, DTT and other low-molecular-weight components were removed using UA buffer (8 M Urea, 150 mM Tris-HCl pH 8.0) by repeated ultrafiltration (Microcon units, 30 kD). Then 100 μl 0.05 M iodoacetamide in UA buffer was added to block reduced cysteine residues and the samples were incubated for 20 min in darkness. The filters were washed with 100 μl UA buffer three times and then 100 μl DS buffer (50 mM triethylammoniumbicarbonate at pH 8.5) twice. Finally, the protein suspensions were digested with 2 μg trypsin (Promega) in 40 μl DS buffer overnight at 37°C, and the resulting peptides were collected as a filtrate. The peptide content was estimated by UV light spectral density at 280 nm using an extinctions coefficient of 1.1 of 0.1% (g/l) solution that was calculated on the basis of the frequency of tryptophan and tyrosine in vertebrate proteins. For labeling, each iTRAQ reagent was dissolved in 70 μl of ethanol and added to the respective peptide mixture. The samples were labeled as (TetSBP1)-114, (Act)-115, and were multiplexed and vacuum dried.

### Liquid chromatography (LC)—electrospray ionization (ESI) tandem MS (MS/MS) analysis by Q exactive

The peptide mixture was desalted on C18 Cartridges (Sigma), then concentrated by vacuum centrifugation and reconstituted in 40 μl of 0.1% (v/v) trifluoroacetic acid. MS experiments were performed on a Q Exactive mass spectrometer that was coupled to Easy nLC (Thermo Fisher Scientific). 10 μl of the sample was injected for nanoLC-MS/MS analysis. The peptide mixture (5 μg) was loaded onto a the C18-reversed phase column (Thermo Scientific Easy Column, 10 cm long, 75 μm inner diameter, 3μm resin) in buffer A (0.1% Formic acid) and separated with a linear gradient of buffer B (80% acetonitrile and 0.1% Formic acid) at a flow rate of 250 nl/min controlled by IntelliFlow technology over 240 min. MS data was acquired using a data-dependent top10 method dynamically choosing the most abundant precursor ions from the survey scan (300–1800 m/z) for HCD fragmentation. Determination of the target value is based on predictive Automatic Gain Control (pAGC). Dynamic exclusion duration was 60 s. Survey scans were acquired at a resolution of 70,000 at m/z 200 and resolution for HCD spectra was set to 17,500 at m/z 200. Normalized collision energy was 30 eV and the underfill ratio, which specifies the minimum percentage of the target value likely to be reached at maximum fill time, was defined as 0.1%. The instrument was run with peptide recognition mode enabled.

### iTRAQ protein identification and quantification

MS/MS spectra were searched using MASCOT engine (Matrix Science, London, UK; version 2.2) embedded into Proteome Discoverer 1.3 (Thermo Electron, San Jose, CA.) against Uniprot Human database (136615 sequences, downloaded on May 7th, 2014) and the decoy database. For protein identification, the following options were used: Peptide mass tolerance = 20 ppm, MS/MS tolerance = 0.1 Da, Enzyme = Trypsin, Missed cleavage = 2, Fixed modification: Carbamidomethyl (C), iTRAQ 4-plex (K), iTRAQ 4-plex (N-term), Variable modification:Oxidation(M), FDR≤0.01. Considering that multiple MS/MS spectra match to one peptide, normalization of the signal intensities of each MS/MS spectra was performed to find the most likely expression ratio for a peptide. For quantitative changes, a 1.2-fold cutoff was set to determine up-accumulated and down-accumulated proteins, with a p-value < 0.05.

### Bioinformatics analysis

Functional analysis of proteins identified was conducted using Gene Ontology (GO) annotation (http://www.geneontology.org/) and proteins were categorized according to their biological process, molecular function and cellular localization [11]. The differentially accumulated proteins were further assigned to the Kyoto Encyclopedia of Genes and Genomes (KEGG) database (http://www.genome.jp/kegg/pathway.html) [12].

## Results

### SBP1 induction in nude mice inhibited tumor growth of xenografts

**Fig 1:**
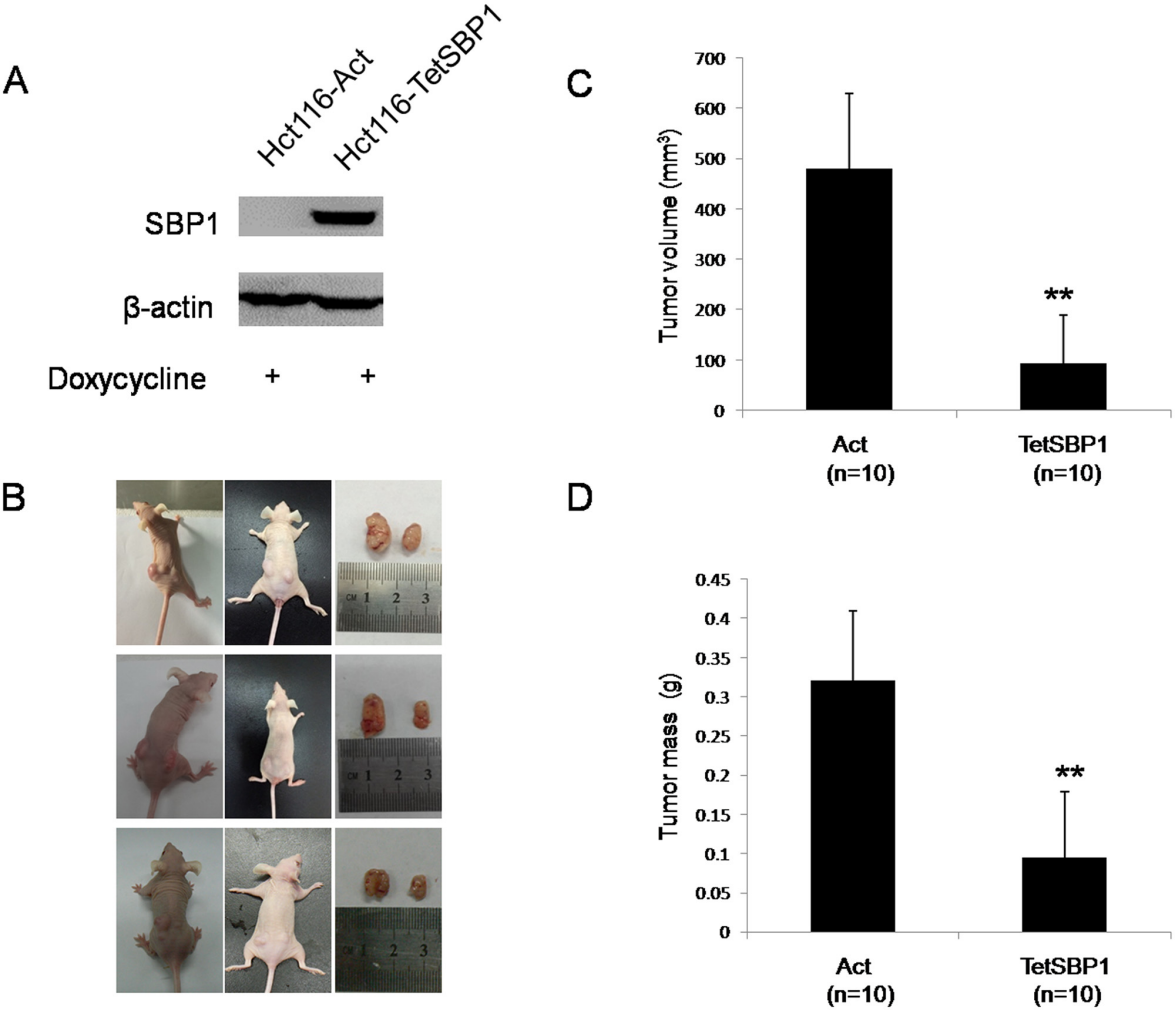
SBP1 induction in nude mice inhibited tumor growth of xenografts. **(A)** SBP1 induced by doxycycline in HCT116-TetSBP1 cells were validated by immunoblot analysis. **(B)** Photographs illustrate representative tumors in xenografts with SBP1 induction (TetSBP1, right) compared to tumors without SBP1 induction (Act, left). 10 mice in total. **(C-D)** SBP1 induction results in a decline of tumor volume and weight. Results were analyzed with Student’s t-test and shown as mean±SD. **P<0.01.

SBP1 expression levels in HCT116-Act and HCT116-TetSBP1 cells were determined by immunoblotting analysis. As shown in Fig 1A, HCT116-TetSBP1 cell line expressed high level of SBP1 after doxycycline induction, compared with the HCT116-Act cells. To test whether SBP1 overexpression had tumor suppressing function *in vivo* as reported previously [13], we performed xenograft experiments in CD1 Nu nude mice (n = 10 in total). HCT116-Act and HCT116-TetSBP1 cells were treated with 1μg/mL doxycycline for 72 hours and then 2×10^6^ cells in 100 μl PBS were injected subcutaneously into 6–8 weeks-old BALB/c CD-1 Nu female mice on the different sides, respectively. HCT116-Act was on the left side and HCT116-TetSBP1 was on the right side. To maintain persistently induction of SBP1, doxycycline (200 μg/mL) dissolved in 5% sucrose supplied as drinking water was exchanged every 3 days. Four weeks after injection, tumors were isolated, and tumor weight (g) and volume (mm^3^) were analyzed. We found that the tumor sizes derived from HCT116-TetSBP1 cells were much smaller than those in the control group HCT116-Act cells (Fig 1B and 1C). Additionally, the tumors weight derived from the HCT116-TetSBP1 cells was significantly reduced, compared to HCT116-Act (Fig 1D). These results strongly suggested that *in vivo* induction of SBP1 showed tumor inhibition in mice.

### SBP1 induction suppressed tumor metastasis *in vivo*

**Fig 2:**
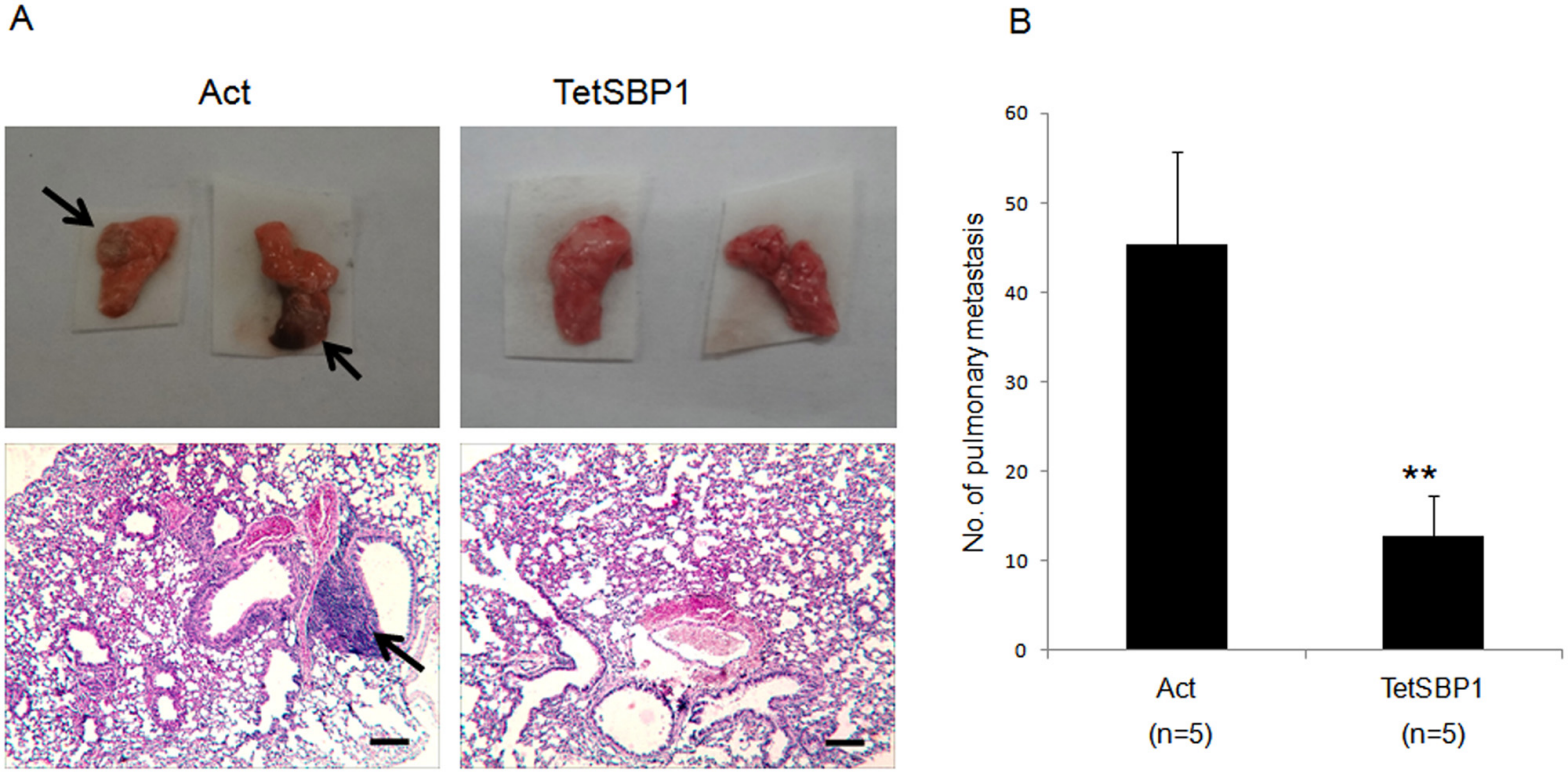
SBP1 induction suppressed tumor metastasis *in vivo* **(A)** Representative images of lungs and hematoxylin and eosin (HE)-staining of lungs isolated from mice that received tail vein injection of HCT116-Act cells (Act) and HCT116-TetSBP1 cells (TetSBP1). Each group contains 5 mice. Arrows illustrates the visible nodules and metastatic nodules in lung. Scale bar = 200 μm. **(B)** SBP1 induction inhibits tumor metastasis *in vivo*. The numbers of pulmonary metastatic nodules were counted under microscope and analyzed with Student’s t-test. Results are shown as mean±SD. **P<0.01.

To examine SBP1 inhibition of tumor metastasis, the HCT116-Act and HCT116-TetSBP1 cells were treated with 1μg/mL doxycycline for 72 hours and then 2×10^6^ cells in 100 μl PBS were injected into the lateral tail veins of 6–8 weeks-old BALB/c CD-1 Nu female mice (n = 5 for each group). To maintain persistently induction of SBP1, doxycycline (200 μg/mL) dissolved in 5% sucrose supplied as drinking water was exchanged every 3 days. After 6 weeks mice were anesthetized and mouse lungs were harvested, fixed and dissected. We found that the mice transplanted with HCT116-Act cells had visible pulmonary metastatic nodules, while no mouse in the HCT116-TetSBP1 group showed visible lung metastasis (Fig 2A). After hematoxylin and eosin (HE) staining, the numbers of pulmonary metastatic nodules were counted. The results showed that mice with SBP1 induction (HCT116-TetSBP1 group) resulted in a significant decrease in pulmonary metastatic nodules (Fig 2B), indicating a metastatic inhibition of SBP1 *in vivo*.

### Quantitative identification and bioinformatics analysis of tumor tissue proteins assayed by iTRAQ

To determine the mechanisms of SBP1-mediated inhibition of tumor growth and metastasis in mice, we extracted total protein from the tumor tissue of HCT116-Act injected (Act) and HCT116-TetSBP1 injected mice (TetSBP1), and protein profiles were conducted using iTRAQ. Ultimately, 9147 unique peptides, 2015 protein groups and 1975 proteins were identified. The distribution of lengths and numbers of peptides, mass and sequence coverage of proteins are provided (S1 Table).

**Fig 3:**
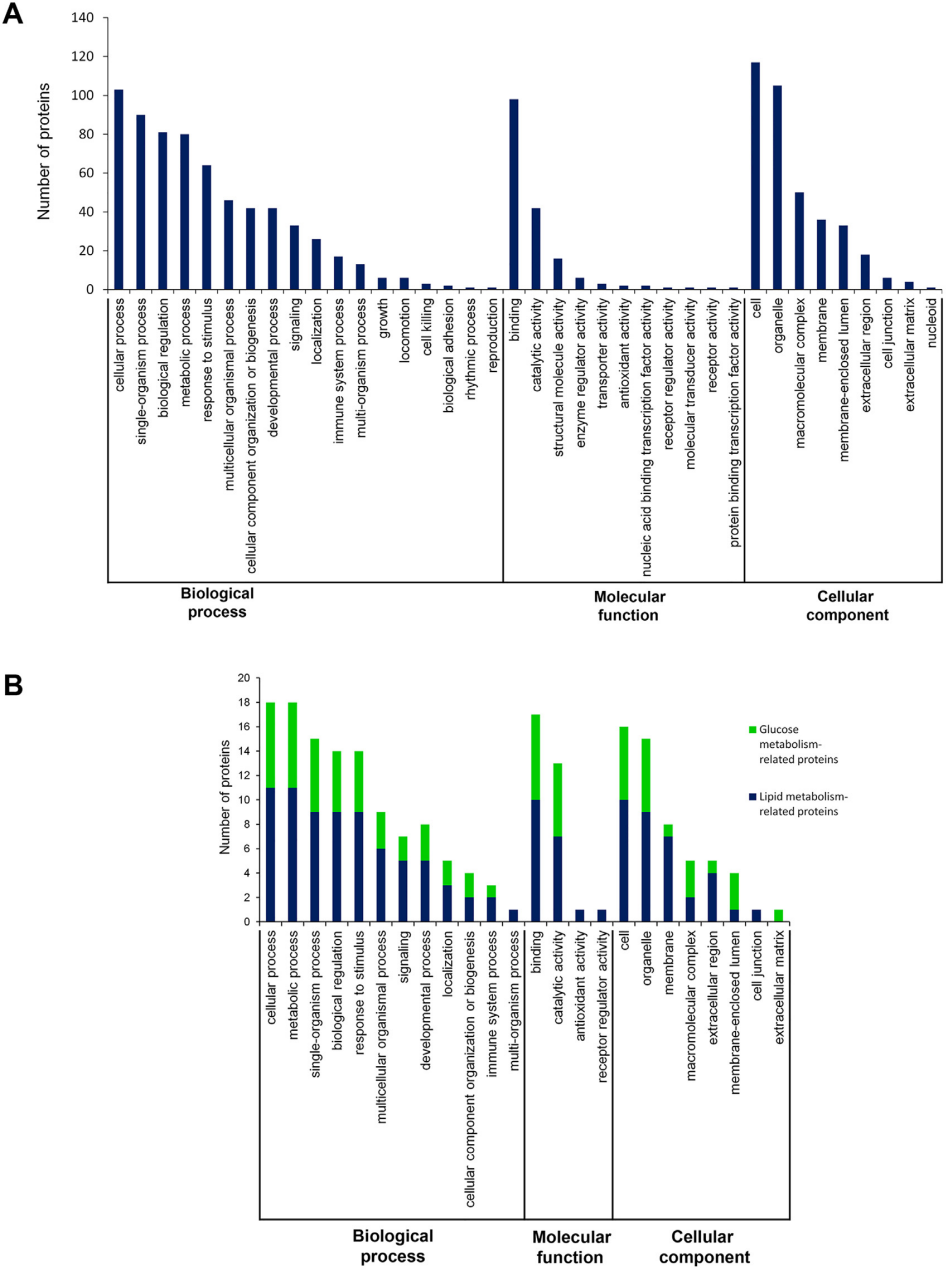
Quantitative identification and bioinformatics analysis of tumor tissue proteins assayed by iTRAQ. **(A)** All 132 differentially accumulated proteins were classified into three groups: biological process, molecular function, and cellular component through GO analysis. **(B)** The numbers of lipid/glucose metabolism-related proteins were shown through GO analysis.

The alterations in the protein profile in response to SBP1 induction were analyzed. 132 proteins showed a significant difference (p-value < 0.05) with an FDR of less than 1%, including 53 upregulated proteins and 79 downregulated proteins. Relevant detailed information are provided (S2 Table). These differentially accumulated proteins enriched 1364 annotations through GO analysis, and were classified into three groups: biological process, molecular function, and cellular component (Fig 3A). The main categories of biological process included cellular process, single-organism process, biological regulation, metabolic process and response to stimulus. Based on different molecular functions, these proteins were divided into the groups of binding, catalytic activity, structural molecule activity, enzyme regulator activity, transporter activity and antioxidant activity. These proteins were also involved in different cellular components, including cell, organelle, macromolecular complex, membrane and membrane-enclosed lumen. These differentially accumulated proteins were further classified using the KEGG database. The major pathways involved in the differentially expressed proteins resulting from SBP1 induction, including transcriptional misregulation in cancer, tight junction, lysosome, protein processing in endoplasmic reticulum, HIF-1 signaling pathway, PI3K-Akt signaling pathway, pathways in cancer, proteoglycans in cancer, microRNAs in cancer, and glycolysis/gluconeogenesis, and other pathways. Recent studies have demonstrated that lipid/glucose metabolism is highly associated with carcinogenesis [14–17]. Therefore, we then analyzed the mostly changed proteins that linked to lipid/glucose metabolism from the mouse xenograft tumors. Interestingly, thirteen lipid metabolism-related proteins and seven glucose metabolism-related proteins were significantly changed by SBP1 induction in mouse xenograft tumors. All these lipid/glucose metabolism-related proteins were illustrated in Fig 3B based on their biological functions through Gene Ontology (GO) analysis.

### Alterations of lipid/glucose metabolism-associated proteins in the response to SBP1 induction

As shown in Fig 3B, thirteen lipid metabolism-related proteins were selected, including five upregulated proteins and eight downregulated proteins (Table 1). The most upregulated proteins by SBP1 induction included DKK1 that inhibits WNT pathway [18], DHCR7 that controls cholesterol and Vitamin D synthesis [19], ANXA4 that inhibits NFkB pathway and IL-8 [20], and AGPAT5 that inhibits breast cancer cell proliferation [21]. All these proteins have been reported to show potential anticancer effects. Whereas, other proteins, such as LPCAT2 that is highly expressed in inflammatory cells [22], BPIFA3 that is highly expressed in lung cancer [23], PPIA that activates NFkB pathway [24], and FABP4 that has pro-angiogenic role [25], were downregulated in SBP1 overexpression xenograft tumors. In contrast, seven glucose metabolism-related proteins were significantly downregulated in SBP1 overexpression xenograft tumors, including GAA, GAPDH, ALDH2, Thioredoxin, ENO3, UGDH and Lumican (Table 2).

**Table 1:** Identified differentially accumulated proteins related to lipid metabolism.

Uniprot Accession	Name	GO-ID	Function	Act/TetSBP1 ratio	p- value
O94907	DKK1 (Dickkopf-related protein 1)	GO:0050750	low-density lipoprotein particle receptor binding	0.47	<0.001
B7Z532	HSP60 (60 kDa heat shock protein, mitochondrial)	GO:0046696	lipopolysaccharide receptor complex	0.51	<0.001
		GO:0001530	lipopolysaccharide binding		
A8K0D2	DHCR7 (7-Dehydrocholesterol reductase)	GO:0006695	cholesterol biosynthetic process	0.80	0.0076
P09525	ANXA4 (Annexin A4)	GO:0005544	calcium-dependent phospholipid binding	0.81	0.0116
H0YC22	AGPAT5 (1-acyl-sn-glycerol-3-phosphate acyltransferase epsilon)	GO:0046474	glycerophospholipid biosynthetic process	0.82	0.0167
Q7L5N7	LPCAT2 (Lysophosphatidylcholine acyltransferase 2)	GO:0046474	glycerophospholipid biosynthetic process	1.21	0.0250
O75715	GPX5 (Epididymal secretory glutathione peroxidase)	GO:0006629	lipid metabolic process	1.22	0.0185
Q5ZEY3	GAPDH (Glyceraldehyde-3-phosphate dehydrogenase)	GO:0005811	lipid particle	1.28	0.0045
F6XY72	NME2 (Nucleoside diphosphate kinase B)	GO:0030027	lamellipodium	1.28	0.0042
B4YAH7	ALDH2 (Aldehyde dehydrogenase, mitochondrial)	GO:0046486	glycerolipid metabolic process	1.28	0.0042
Q9BQP9	BPIFA3 (BPI fold-containing family A member 3)	GO:0008289	lipid binding	1.29	0.0030
Q71V99	PPIA (Peptidyl-prolyl cis-trans isomerase A)	GO:0034389	lipid particle organization	1.30	0.0023
P15090	FABP4 (Fatty acid-binding protein 4)	GO:0046486	glycerolipid metabolic process	1.44	<0.001

**Table 2:** Identified differentially accumulated proteins related to glucose metabolism.

Uniprot Accession	Description	GO-ID	Function	Act/TetSBP1 ratio	p-value
B7Z5V6	GAA (Lysosomal alpha-glucosidase)	GO:0004558	alpha-glucosidase activity	1.24	0.0119
		GO:0017177	glucosidase II complex		
Q5ZEY3	GAPDH (Glyceraldehyde-3-phosphate dehydrogenase)	GO:0006006	glucose metabolic process	1.28	0.0045
B4YAH7	ALDH2 (Aldehyde dehydrogenase, mitochondrial)	GO:0006094	gluconeogenesis	1.28	0.0042
P10599	TXN (Thioredoxin)	GO:0006662	glycerol ether metabolic process	1.29	0.0029
P13929	ENO3 (Beta-enolase)	GO:0006094	gluconeogenesis	1.29	0.0029
O60701	UGDH (UDP-glucose 6-dehydrogenase)	GO:0003979	UDP-glucose 6-dehydrogenase activity	1.31	0.0018
P51884	LUM (Lumican)	GO:0006027	glycosaminoglycan catabolic process	1.40	<0.001

To validate the differential alterations of the lipid/glucose metabolism-related proteins assayed from iTRAQ proteomic analysis in mouse xenograft tumors, immunoblotting analyses were performed. Consistent with those observed from iTRAQ profile, two proteins (DKK1 and ANX4) were significantly increased and four proteins (PPIA, FABP4, ALDH2 and UGDH) were significantly decreased in SBP1 overexpression xenograft tumors (S1 Fig). The high consistency indicated the high reliability of the iTRAQ results. MS/MS data of these six proteins are provided (S2 Fig).

### Tumor inhibition of SBP1 was associated with multiple signaling pathways *in vivo*

**Fig 4:**
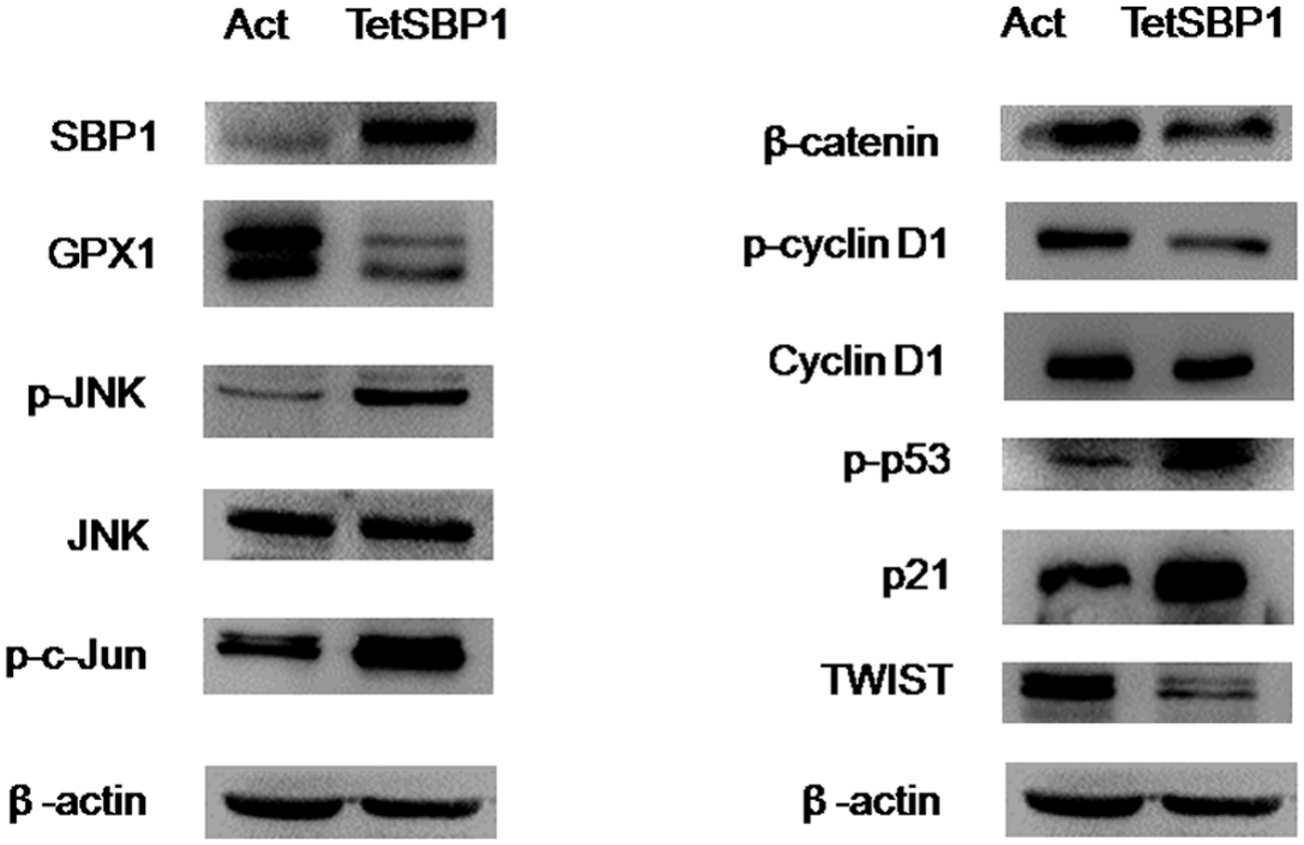
Tumor inhibition of SBP1 was associated with multiple signaling pathways *in vivo*. Several typical markers of different signaling pathways were examined using iTRAQ proteomic analysis protein samples. These cancer related signaling pathways respond differently to SBP1 induction.

To determine the additional mechanisms of SBP1 induction mediated inhibition of tumor growth and metastasis, we analyzed tumor growth and metastasis-associated signaling pathways using mouse xenograft tumor protein samples which were also used for iTRAQ proteomic analysis. First, GPX1 was downregulated in TetSBP1 tumors (Fig 4), consistent with those observed in human prostate cancer tissues [26], but it was different from the *in vitro* studies which showed that overexpressing SBP1 did not affect GPX1 protein levels in HCT116 cells although GPX1 activities were significantly downregulated [6]. This finding again suggested inverse correlation between SBP1 and GPX1 *in vivo* not *in vitro*. Second, induction of SBP1 led to activation of JNK1/Wnt pathway, in terms of increased phosphorylation of JNK1 and c-Jun, and decreased expression of beta-catenin and its downstream targets Cyclin D1, in particular, phosphorylated Cyclin D1 was significantly downregulated (Fig 4). In addition, beta-catenin inhibitors p53 (phosphorylated p53, p-p53) and p21, a direct downstream target of p53, were increased in response to SBP1 induction in mouse TetSBP1 tumors (Fig 4). Third, the induction of SBP1 led to a significant reduction of TWIST (Fig 4), a critical regulator for EMT and metastasis.

## Discussion

Reduced expression of SBP1 has been observed in several types of human cancers, including colorectal, lung, esophageal, gastric, breast and liver cancers, and the reduction of SBP1 is associated with poor survival [5]. Decrease of SBP1 increased cell motility, promoted cell proliferation, and suppressed cell differentiation *in vitro*, although the mice with SBP1 genetic deficiency (i.e. SBP1 knockout mice) did not show obvious phenotypes [3,27,28]. In contrast, increasing SBP1 expression in cells by transfection of SBP1-expressing plasmid could leads to senescence, inhibition of cancer cell proliferation, migration and tumor growth in immunocompromised mice [3,13,29,30]. Our previous studies have shown that SBP1 was decreased in most colorectal tumors compared to nontumor tissues whereas it has variable expression levels in different cancer cell lines [6,13,29]. For example, SBP1 is highly expressed in LS174T, Caco-2, HT-29 and MCF-7, but undetectable in HCT116. So human colon cancer cell line HCT116 was used to overexpress SBP1 in our study. In this study, using an inducible system and mouse xenograft model, we have found that *in vivo* induction of SBP1 showed significant anti-cancer effects, including inhibited tumor growth and metastasis in nude mice. Advantages of using this SBP1 Tet-on inducible gene expression system are listed as following: 1) This inducible gene expression system makes SBP1 overexpression controllable, thus, the cells are maintained without SBP1 interference and the transfection damage can be minimized when overexpression is needed; 2) In this study, SBP1 expression has no tag modification and the expressed protein stays in natural state. All of these observation suggested that both endogenous and ectopic SBP1 could serve as a potential tumor suppressor.

To determine the underlying molecular mechanisms, the proteomic technique iTRAQ label assay was employed. Among the 1975 identified proteins, 132 proteins were differentially expressed in SBP1-induced tumor tissue. Further analysis narrowed down the differentially expressed proteins into thirteen lipid metabolism-related and seven glucose metabolism-related proteins. Among the thirteen lipid metabolism-related proteins, five were upregulated (DKK1, HSP60, DHCR7, ANXA4 and AGPAT5) and eight were downregulated (LPCAT2, GPX5, GAPDH, NME2, ALDH2, BPIFA3, PPIA and FABP4). In the upregulated proteins by SBP1 induction, DKK1 is known as an inhibitor of the Wnt/β-catenin pathway [18]. Upregulation of DKK1 could active Jun N-terminal kinase (JNK) and induce apoptosis [31,32], and may negatively regulate EMT through TWIST inhibition [33]. In fact, our previous studies have demonstrated that activated JNK1 can also negatively regulate β-catenin through GSK3-beta [34]. In addition, the increased expression of DKK1 could lead to downregulation of Cyclin D1, a direct downstream target of beta-catenin. Our previous work has also demonstrated a regulatory interaction between JNK1 and p21 [35]. Herein we found that as a response to the upregulation of DKK1 and JNK1, p21 and its upstream target p53 were also increased (Fig 4). Interestingly, in the Wnt/beta-catenin signal pathway, there is a negative regulatory interaction between beta-catenin/Cyclin D1 and p53/p21 [36]. Among the validated lipid metabolism-related proteins, ANXA4 was upregulated and PPIA was downregulated. It has been reported that ANXA4 could suppresses NF-kB transcriptional activity by interacting with the NF-kB p50 subunit [20] and cytokines (e.g. IL-8 [37]). In contrast, PPIA could active NF-kB pathway and inflammatory signaling [24]. Numerous studies have strongly shown that activation of NF-kB and chronic inflammation play crucial roles in cancer formation and progression [38]. Another downregulated protein was FABP4. It has been reported that FABP4 mediates VEGFA-dependent pro-angiogenic effects, which is linked to NOTCH signaling during carcinogenesis and metastasis [25].

All seven glucose metabolism-related proteins were downregulated in SBP1 induced-tumor tissue, including GAA, GAPDH, ALDH2, Thioredoxin, ENO3, UGDH and Lumican. UGDH (UDP-glucose dehydrogenase) catalyzes oxidation of UDP-glucose to yield UDP-glucuronic acid, a precursor of hyaluronic acid (HA) and other glycosaminoglycans (GAGs) in extracellular matrix. A previous study has demonstrated that decreasing UGDH inhibits cell aggregation and migration [39]. Furthermore, it was reported that ALDH2 inactivation could increase cytotoxicity produced by lipid peroxidation [40]. These results suggested that downregulation of UGDH and ALDH2 might be attributable to the anticancer effects of SBP1.

**Fig 5:**
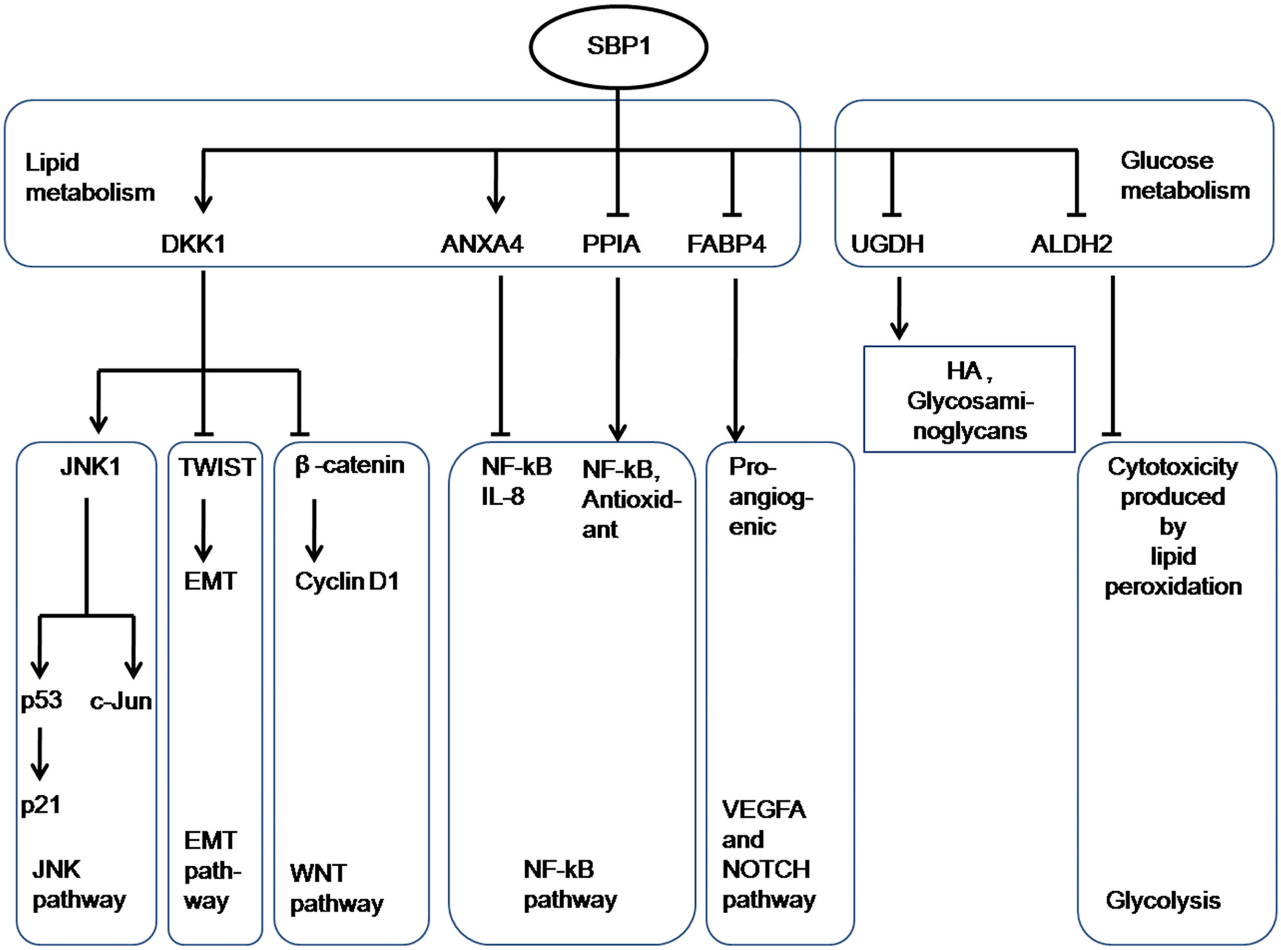
Hypothetic pathways of SBP1-mediated anti-cancer functions *in vivo*. SBP1-mediated anti-cancer effects may be through lipid/glucose metabolism. The possible functional regulation between SBP1 and related proteins is illustrated.

In summary, by using a SBP1 inducible system and *in vivo* mouse model, our studies have clearly shown that tumor inhibition roles of SBP1 are associated with alterations of lipid/glucose metabolism-related proteins through the involvement of multiple signaling pathways, revealing a novel molecular mechanism of tumor inhibition by SBP1. The possible functional relationship between SBP1 and its target proteins is illustrated in Fig 5. It should be noted that the changes of these proteins by SBP1 induction could be caused by one or more of the lipid/glucose metabolism-related proteins, such as DKK1, ANXA4 or NF-kB, because any one of these molecules plays critical role in carcinogenesis and progression through regulating downstream targets. Whether it is true and how these changed proteins interact and regulate each other is not clear and under determination. More studies on SBP1 anti-cancer function should especially pay attention to these lipid/glucose metabolism-related proteins, and protein-protein interaction and synergistic functions of these proteins needs further investigation.

## Supporting Information

S1 Table All peptides and proteins identified by iTRAQ. (XLSX)

S2 Table Detailed information of differentially accumulated proteins in response to SBP1 induction. (XLSX)

S1 Fig Validation of iTRAQ proteomic results by western blot analyses. (TIF)

S2 Fig MS/MS data of six valided proteins. (PPTX)
