# Supplementary figures and images for: Quantitative Proteomic Analysis Reveals That Anti-Cancer Effects of Selenium-Binding Protein 1 In Vivo Are Associated with Metabolic Pathways
Source: PLoS One. 2015 May 14;10(5):e0126285. doi: 10.1371/journal.pone.0126285 (PMC4431778; doi:10.1371/journal.pone.0126285)

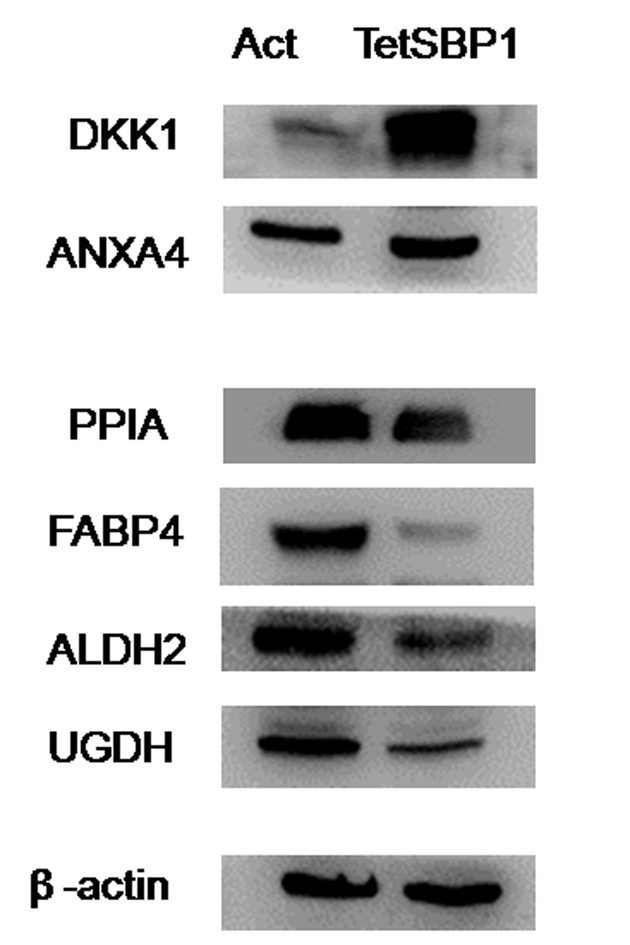

Supplement: S1 Fig — (TIF) [file pone.0126285.s003.tif]
