# Supplementary material for: Quantitative Proteomic Analysis Reveals That Anti-Cancer Effects of Selenium-Binding Protein 1 In Vivo Are Associated with Metabolic Pathways
Source: PLoS One. 2015 May 14;10(5):e0126285. doi: 10.1371/journal.pone.0126285 (PMC4431778; doi:10.1371/journal.pone.0126285)
Supplement: S2 Fig — (PPTX) [file pone.0126285.s004.pptx]

## Slide 1
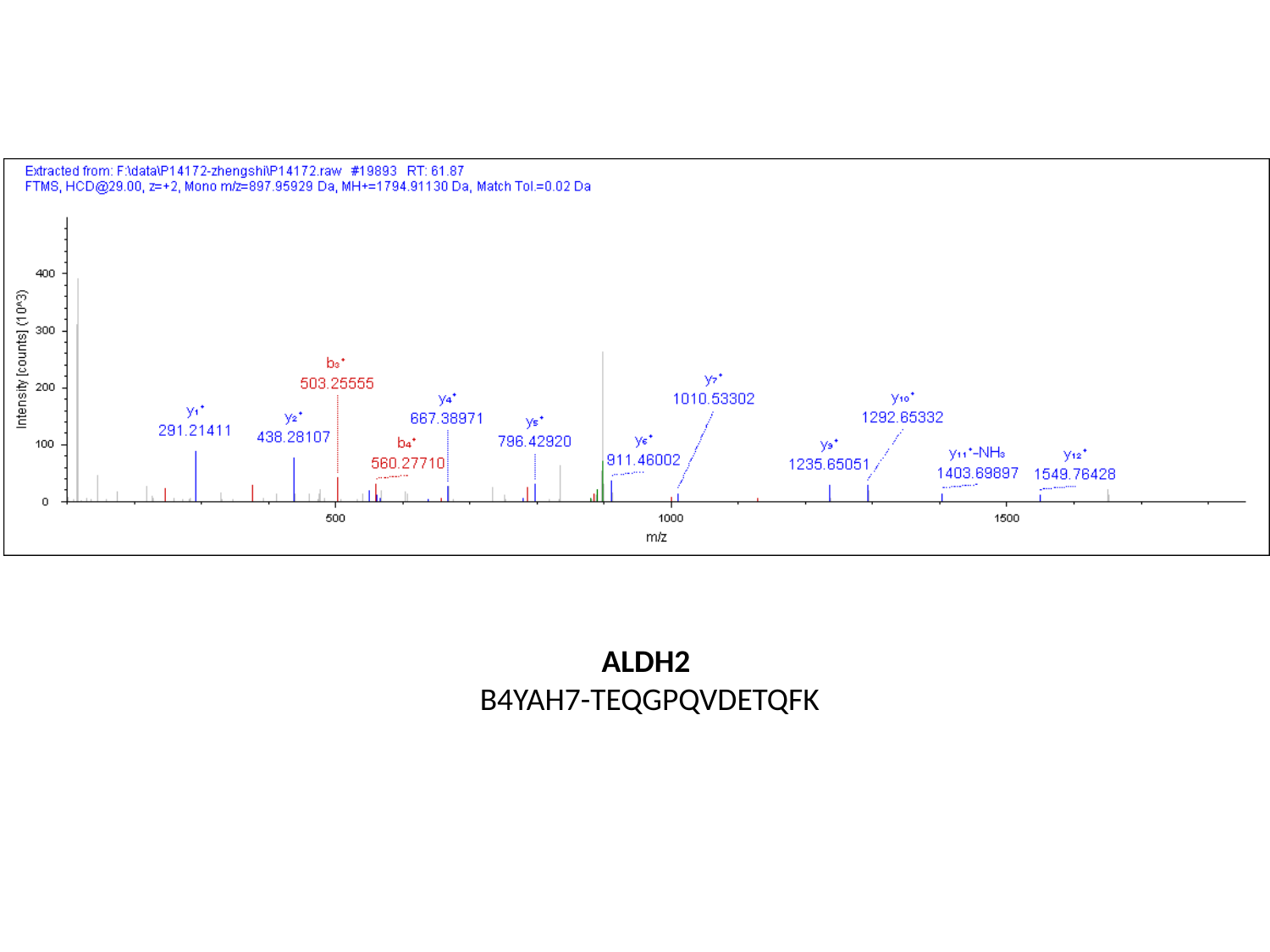

ALDH2
B4YAH7-TEQGPQVDETQFK

## Slide 2
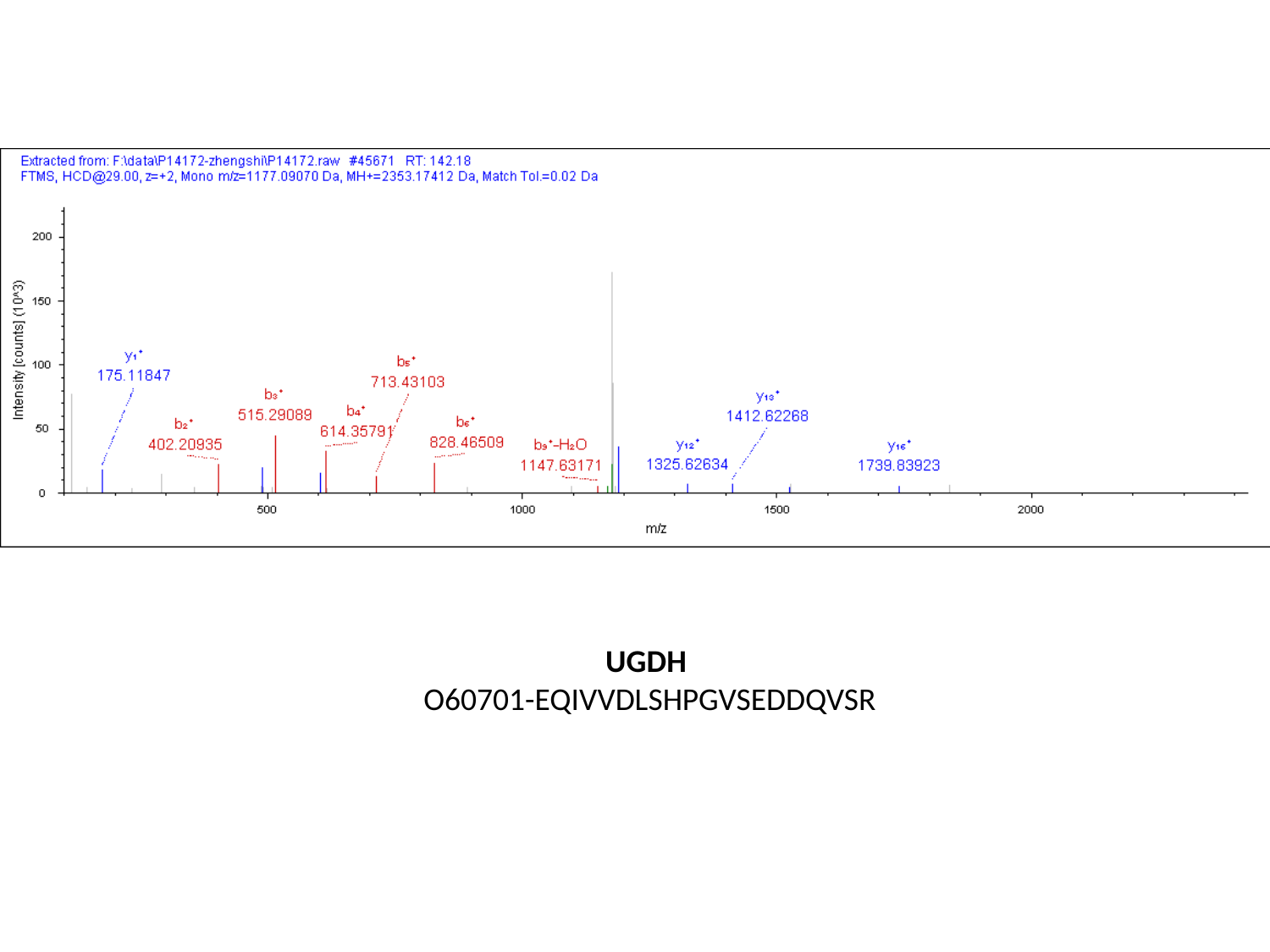

UGDH
O60701-EQIVVDLSHPGVSEDDQVSR

## Slide 3
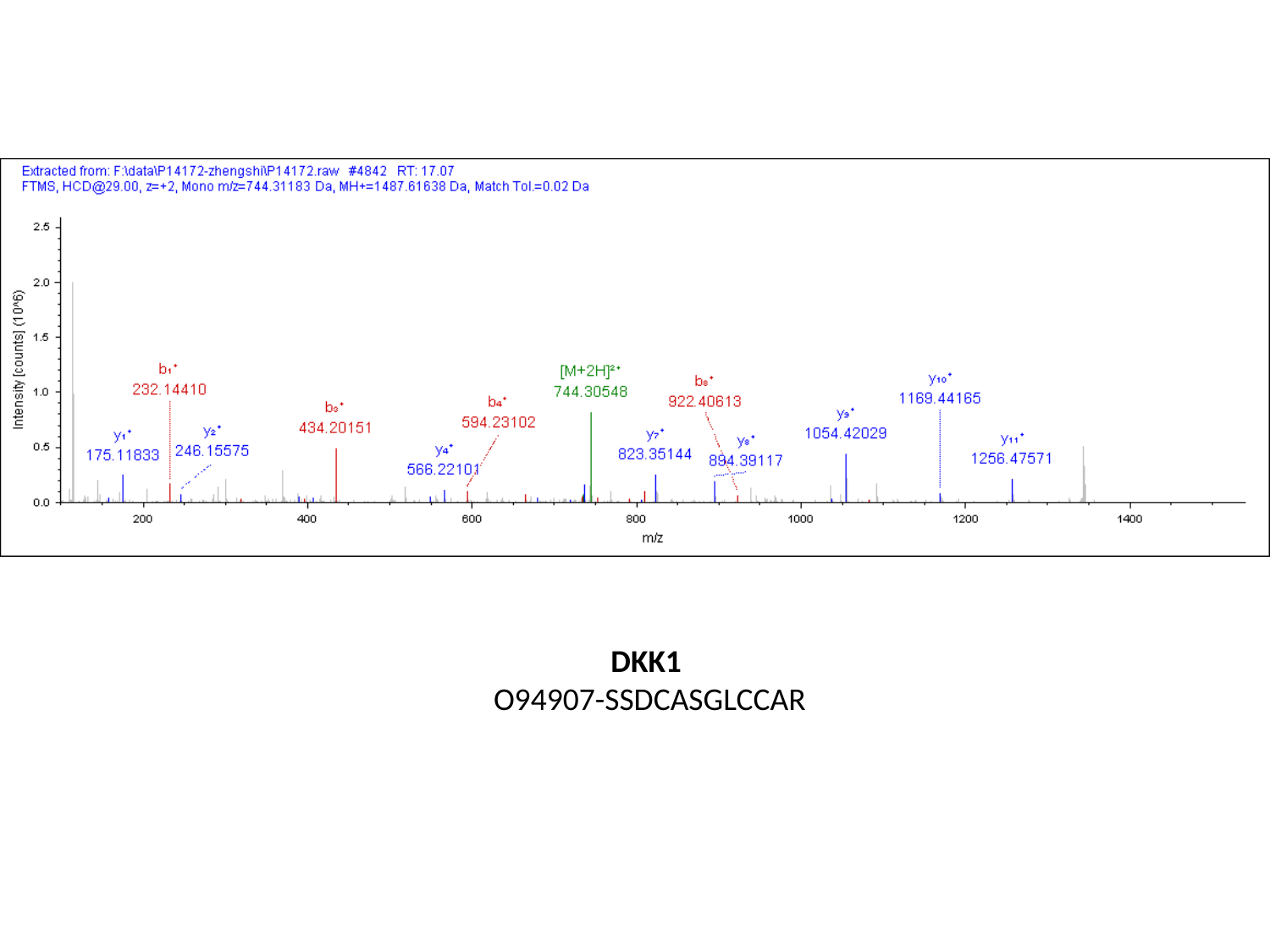

DKK1
O94907-SSDCASGLCCAR

## Slide 4
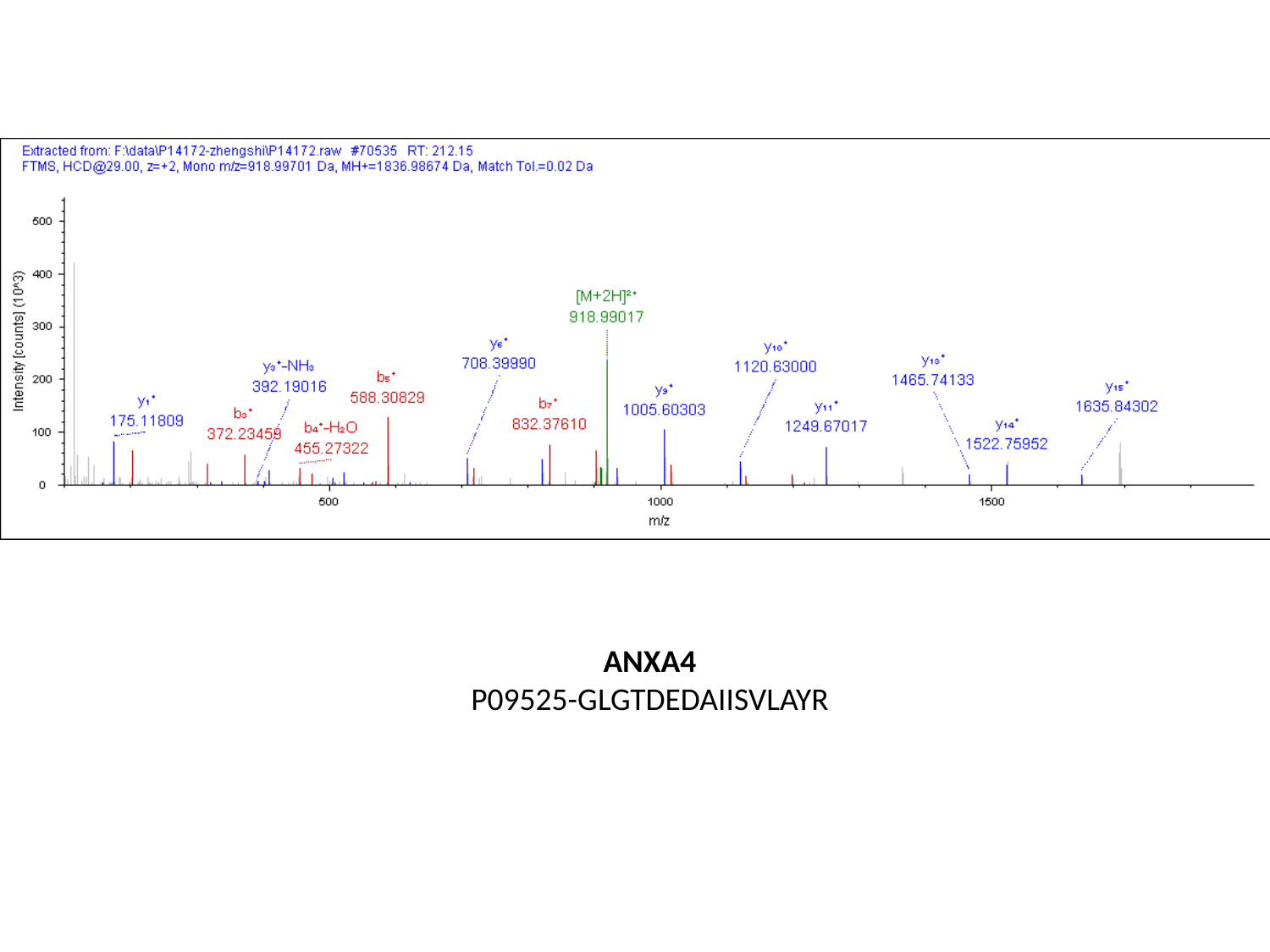

ANXA4
P09525-GLGTDEDAIISVLAYR

## Slide 5
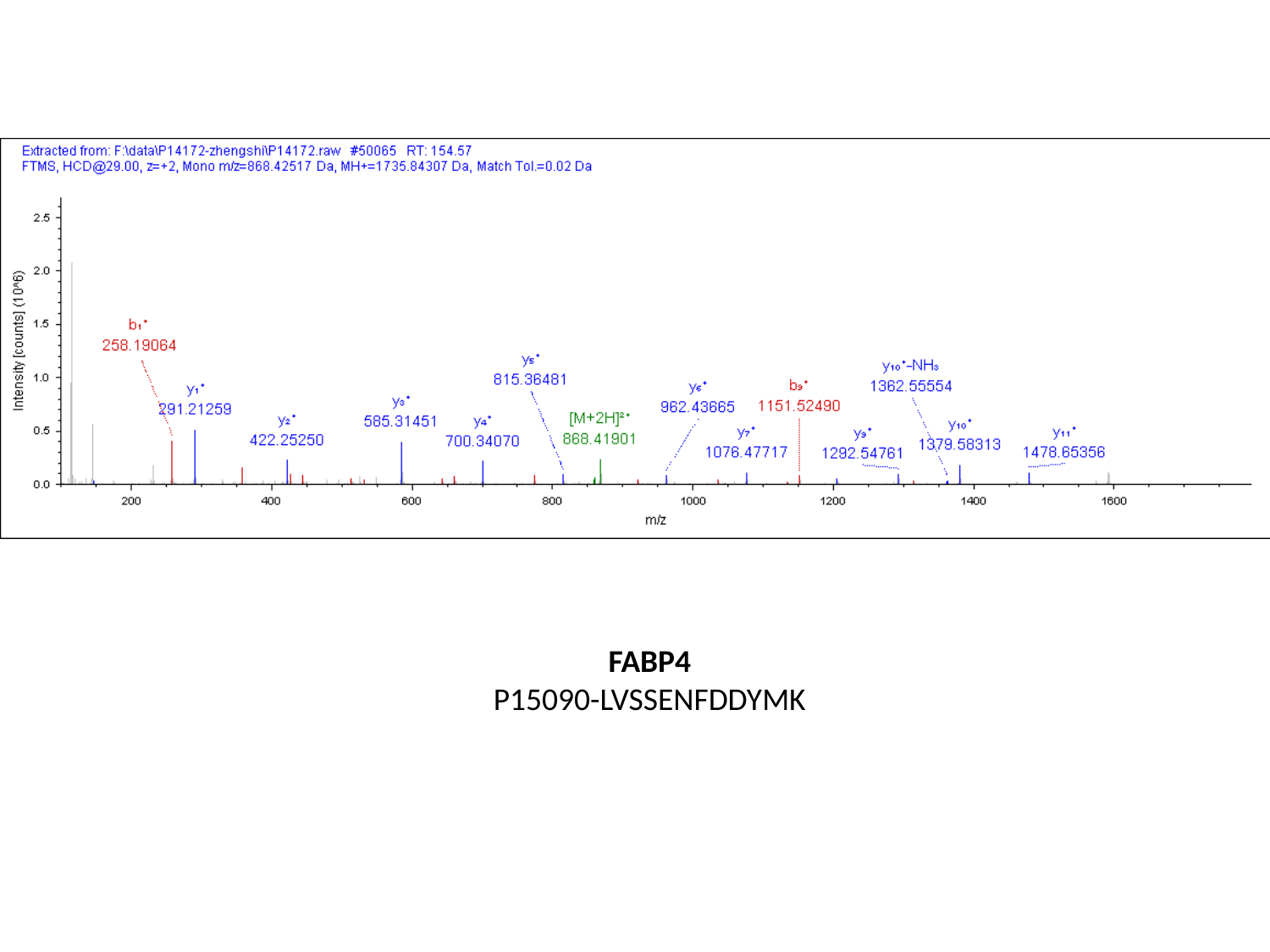

FABP4
P15090-LVSSENFDDYMK

## Slide 6
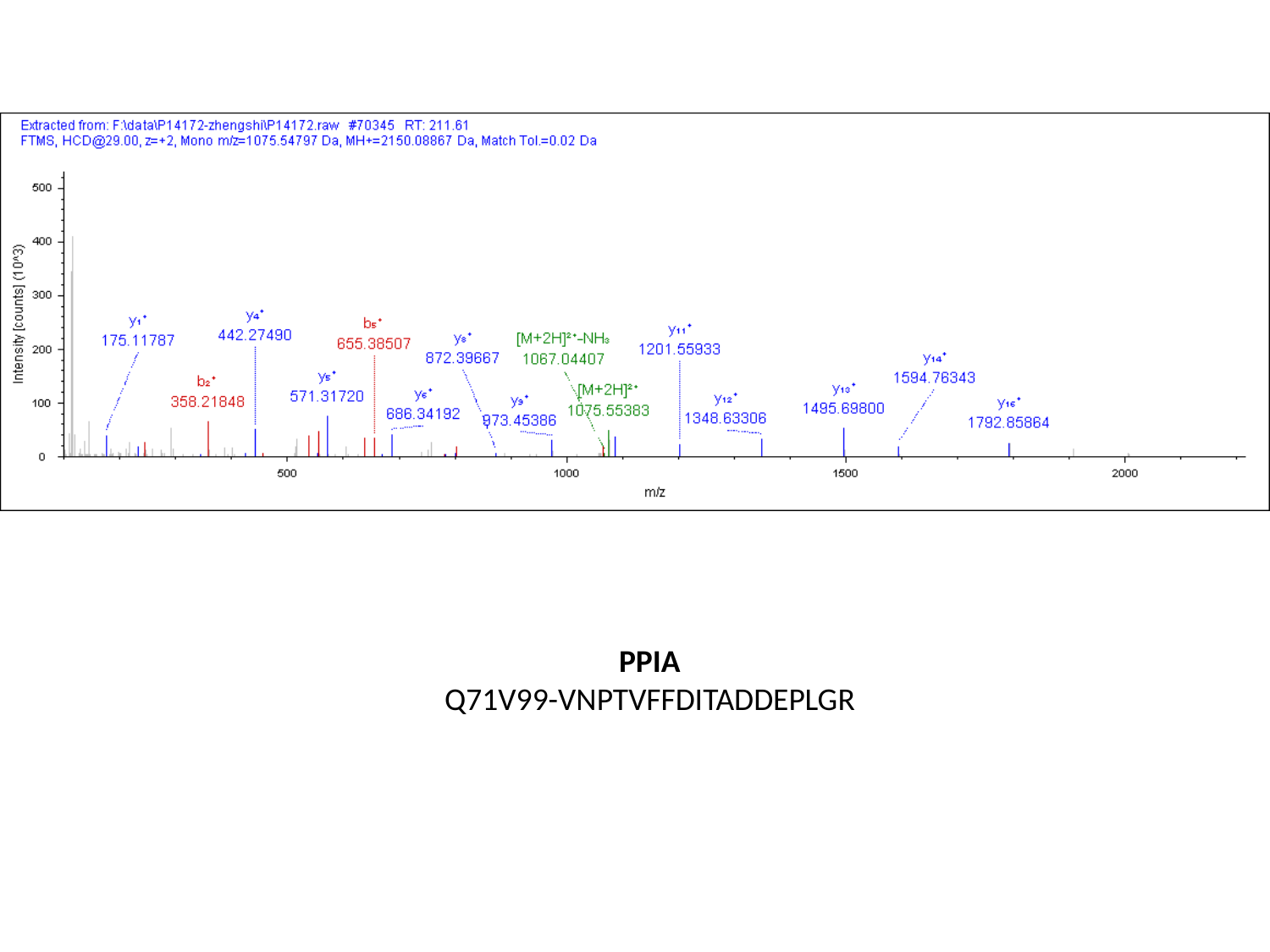

PPIA
Q71V99-VNPTVFFDITADDEPLGR
